# Supplementary material for: An intermetallic molecular nanomagnet with the lanthanide coordinated only by transition metals
Source: Nat Commun. 2022 Apr 19;13:2014. doi: 10.1038/s41467-022-29624-7 (PMC9018761; doi:10.1038/s41467-022-29624-7)
Supplement: Supplementary file 1 — Supplementary Information [file 41467_2022_29624_MOESM1_ESM.pdf]

Supplementary Information

An Intermetallic Molecular Nanomagnet with the Lanthanide Coordinated Only by Transition Metals

Michał Magott,[1] *Maria Brzozowska,*[1] *Stanisław Baran,*[2] *Veacheslav Vieru,*[3] *Dawid Pinkowicz*[1,*]

[1] Faculty of Chemistry, Jagiellonian University, Gronostajowa 2, 30-387 Kraków, Poland
[2] Marian Smoluchowski Institute of Physics, Jagiellonian University, Łojasiewicza 11, 30-348 Kraków, Poland
[3] Maastricht Science Programme, Faculty of Science and Engineering, Maastricht University, Paul-Henri Spaaklaan 1, 6229 EN Maastricht, The Netherlands
*Correspondence: dawid.pinkowicz@uj.edu.pl

Additional experimental and computational details

Details of single-crystal X-ray diffraction (SCXRD)

SCXRD measurements were performed using Bruker D8 Quest Eco diffractometer equipped with Photon II CPAD detector, MoKα sealed tube radiation source and Triumph® optics. The SCXRD experimental details can be found in Supplementary Table 1 and in the respective crystallographic information files (cif) deposited with the Cambridge Crystallographic Data Centre accessible free of charge via www.ccdc.cam.ac.uk/data_request/cif: CCDC 2065530 (**ErRe$_3$** at 100 K), CCDC 2065531 (**ErRe$_3$** at 270 K) and CCDC 2027573 ([Cp_2ReH] at 100 K). The single crystals were removed from the mother liquor directly into the Type NVH Cargille Immersion Oil and mounted using a 100 µm MiTeGen cryoloop. The data collections were performed at 100 K for **ErRe$_3$** and [Cp_2ReH] and then again for **ErRe$_3$** at 270 K for comparison with the experimental PXRD pattern of the bulk sample of **ErRe$_3$**. Data processing was performed using the Apex4 suite of programs – the details can be found in the cif files. The structures were solved using direct methods and refined anisotropically (weighted full-matrix least-squares on F^2, refs. 1 and 2). Hydrogen atoms were placed in the calculated positions and refined as riding on the parent atoms. Structural diagrams were prepared using Mercury 2020.2.0 software (CCDC) and CorelDRAW 2019. The cif file for **ErRe$_3$** collected at 100 K (CCDC 2065530) shows A and B alerts (PLAT971 and PLAT972) related to the "Larger than expected residual density maximum outside metal atom locations" in a close proximity to the heavy metal centers Re and Er. These alerts might be caused by a slight twinning of the crystal which was not included in the data processing and refinement. Noteworthy, if the diffraction data is limited to 0.84 Å resolution and refined again, these alerts disappear – please see the cif file for the same crystal measured at 270 K (CCDC 2065531) with lower resolution up to 0.84 Å due to the higher data collection temperature – this file is almost free of the relevant checkcif alerts (only one B-level alert PLAT972). Additionally, the B-level alert PLAT315 in the structural model of **ErRe$_3$** collected at 100 K (Singly bonded atom detected) is associated with the disorder of the benzene molecule located in the special position.

Details of powder X-ray diffraction (PXRD)

PXRD pattern for **ErRe$_3$** was collected using Bruker D8 Advance Eco diffractometer equipped with Lynxeye silicon strip detector, Cu sealed tube radiation source and capillary stage. A sample of **ErRe$_3$** was ground to a fine powder using an agate mortar inside the glovebox and loaded into a glass capillary 0.3 mm in diameter. The capillary was broken in half inside the glovebox and the open end was sealed using silicon

grease before it was moved to the PXRD instrument and mounted on the goniometer head using bee wax. The PXRD pattern was collected in 27 consecutive scans (12 minutes each) in the 3-50 2ϑ range to exclude the possibility of the decomposition of the sample. No signs of decomposition within the experiment time were observed: the first and the last scans are identical. The experimental PXRD pattern is presented in Supplementary Fig. 4 (red line) and compared against the simulated curve (gray line) obtained from the SCXRD data collected at 270 K (the simulated curves, that include the CuKβ component were exported using Mercury 2020.2.0 software).

Details of infrared spectroscopy (IR)

IR spectra were collected using Nicolet iN10 MX FT-IR microscope in the transmission mode. A small powdered sample of **$ErRe_3$** was placed on the surface of a vacuum-dried (24 h) BaF_2 optical window and sealed inside a vacuum-dried (24 h) Linkam THMS350V temperature-controlled stage. All these operations were performed inside the glovebox and the IR spectra were recorded after removing the stage from the glovebox. The stage was used to protect the sample from decomposition and not for temperature stabilization.

Description of magnetic measurements

Magnetic measurements were performed using Quantum Design MPMS3 Evercool SQUID magnetometer in the magnetic fields up to 7 T for a powdered sample of **$ErRe_3$** placed under a small quantity of anhydrous benzene in a flame-sealed borosilicate tube. The details of the experimental setup were described by some of us previously (ref. 3). The presence of benzene immobilizes the sample below its freezing point of 278 K. The experimental magnetic data were corrected for the diamagnetism of the sample, benzene and the sample holder. Additional AC magnetic susceptibility data in the 10-10 000 Hz range were recorded using Quantum Design PPMS instrument using exactly the same sample.

Details of *ab initio* calculations

To investigate the electronic structure of the compound under study and its magnetic properties, a*b initio* calculations have been carried out with OpenMolcas 19.11 version (ref. 4). The Cholesky decomposition with the threshold of $1.0 \cdot 10^{-7}$ was employed to save disk space. First, a Complete Active Space Self-Consistent Field (CASSCF) calculation (ref. 5) was run by considering eleven electrons spanned by seven 4*f*-type orbitals. Two different basis sets were considered (Supplementary Table 5) and both basis sets predict similar energies and values for the *g*-tensors, that give confidence that a much larger, though computationally more expensive basis set, would not change the results significantly. The spin-orbit coupling was introduced within the RASSI module (ref. 6) where all quartet states (35 in total) and all doublet states (112 in total) were admixed by the spin-orbit coupling. Then, magnetic properties were calculated based on the spin-orbital states within the SINGLE_ANISO module (refs. 4, 7 and 8).

The calculated spin-orbit energies (Supplementary Table 6) demonstrate that the ground Kramers Doublet is well separated from the excited states and shows small transversal components of the *g*-tensor as compared to a very large axial one. This implies that the quantum tunneling of magnetization is suppressed and the relaxation of the magnetization occurs via the excited states. Interestingly, the main magnetic axis is oriented perpendicular to the plane formed by the trigonal Re_3 ligand field (Figure 1C). The relaxation path of the magnetization blocking (Figure 2B) was built as described elsewhere (ref. S9) using SINGLE_ANISO program.

Supplementary Table 1. Selected crystallographic parameters for **$ErRe_3$** at 100 and 270 K and the starting material [Cp_2ReH] at 100 K.

Compound	**$ErRe_3$** (@100 K)	**$ErRe_3$** (@270 K)	[Cp_2ReH]
T / K	100(2)	270(2)	100(1)
CCDC deposition number	2065530	2065531	2027573
Crystal system	trigonal	trigonal	monoclinic
Space group	*R*-3	*R*-3	*P*n
a / Å	41.1853(10)	41.6196(10)	5.8261(4)
b / Å	41.1853(10)	41.6196(10)	9.9522(6)
c / Å	7.6318(3)	7.6943(3)	13.7335(8)
α / °	90	90	90
β / °	90	90	96.651(2)
γ / °	120	120	90
V/Å^3	11210.9(7)	11542.4(7)	790.94(9)
Z	6	6	4
ρ_{calc} / g cm^{-3}	3.009	2.901	2.657
μ / mm^{-1}	17.882	17.367	15.284
F(000)	9168	9090	580
Crystal size / mm^3	0.120 x 0.030 x 0.020	0.120 x 0.030 x 0.020	0.210 x 0.170 x 0.060
Instrument	Bruker D8 Quest Eco	Bruker D8 Quest Eco	Bruker D8 Quest Eco
Radiation	Mo Kα (λ = 0.71073 Å)	Mo Kα (λ = 0.71073 Å)	Mo Kα (λ = 0.71073 Å)
2θ range/°	2.62-33.24	2.59-25.40	2.99-32.72
Reflections collected	46432	112127	2988
Independent reflections	9537	4720	2988
R_{int}	0.0555	0.0845	0.0356
restrains/parameters	6/328	0/310	122/200
$R[F_o > 2\sigma(F_o)]$	0.0543	0.0410	0.0339
$wR(F^2)$	0.1202	0.0793	0.0892
GOF on F^2	1.082	1.128	1.096
$\Delta\rho_{max}$, $\Delta\rho_{min}$ / e·Å^{-3}	4.022, -5.077	1.491, -2.548	2.216, -3.478
Completeness / %	99.5	99.8	99.5

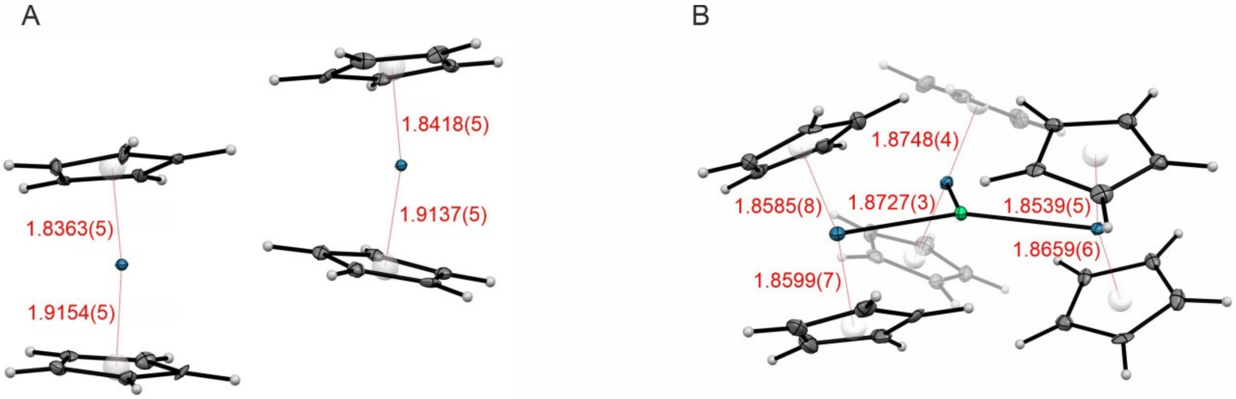

Supplementary Fig. 1. Structural diagrams showing the asymmetric units (ASUs) of the starting material [Cp_2ReH] (A) and the target compound **$ErRe_3$** (B). The red numbers indicate the distances (Å) between the Re atoms and the centroids of the cyclopentadienyl rings. C – grey, H – light grey, Re – blue, Er – green, centroids – white; ellipsoids at the 40 % probability level for A and 30% for B.

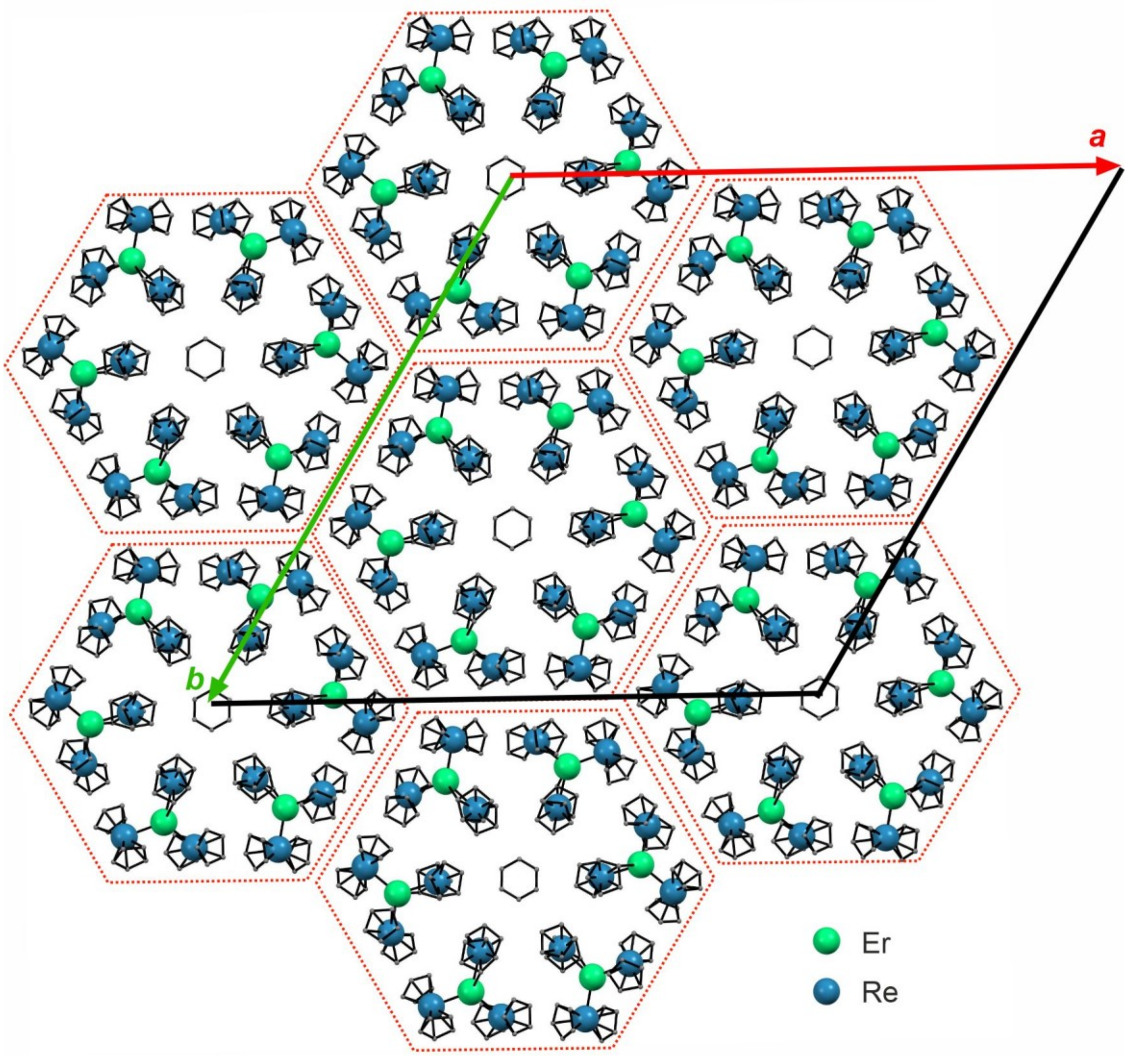

Supplementary Fig. 2. Structural diagram of **$ErRe_3$** as viewed along the *c* crystallographic direction showing the hexagonal packing pattern of the $[Er^{III}(Re^{I}Cp_2)_3]$ molecules highlighted by red dotted hexagons. This type of packing leads to the formation of channels along the *c* axis filled with benzene molecules (crystallization solvent). Cyclopentadienyl ligands and benzene molecules are represented by black sticks and H-atoms are omitted for clarity.

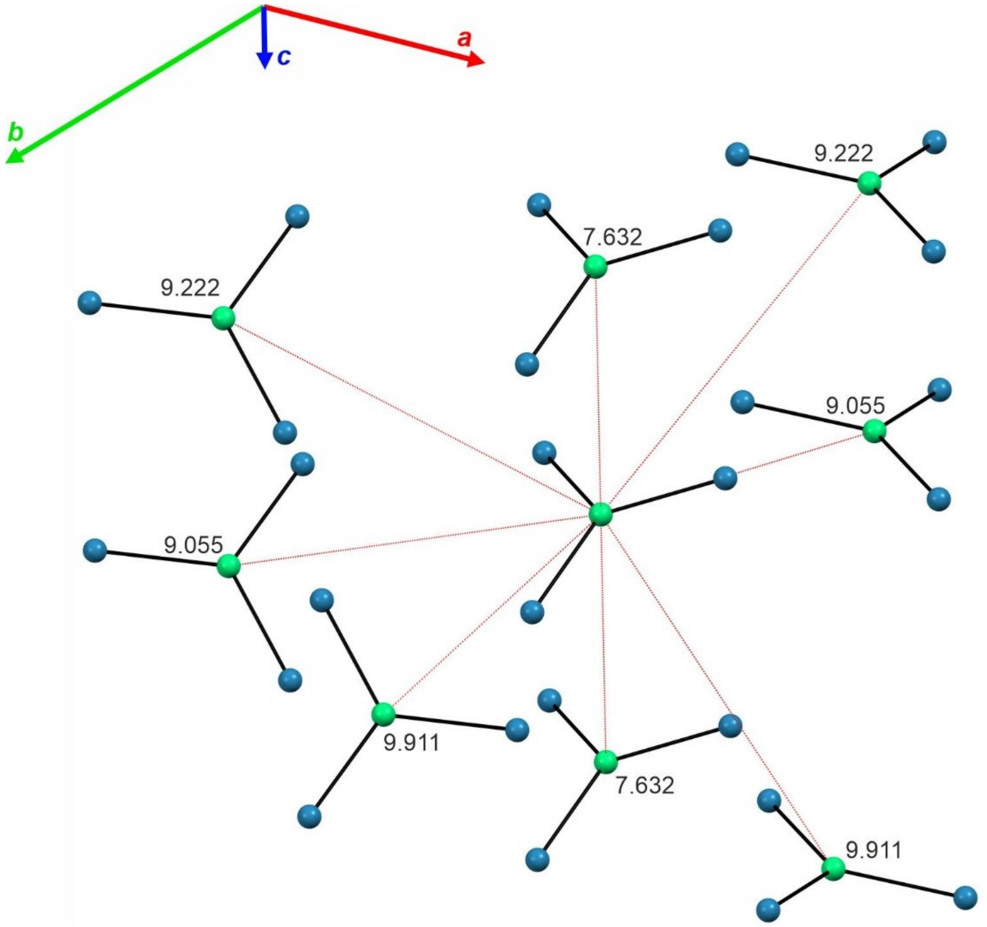

Supplementary Fig. 3. Structural diagram demonstrating and listing Er···Er distances between all **$ErRe_3$** nearest neighbours. The shortest distance is 7.6318(6) Å along the *c* crystallographic direction.

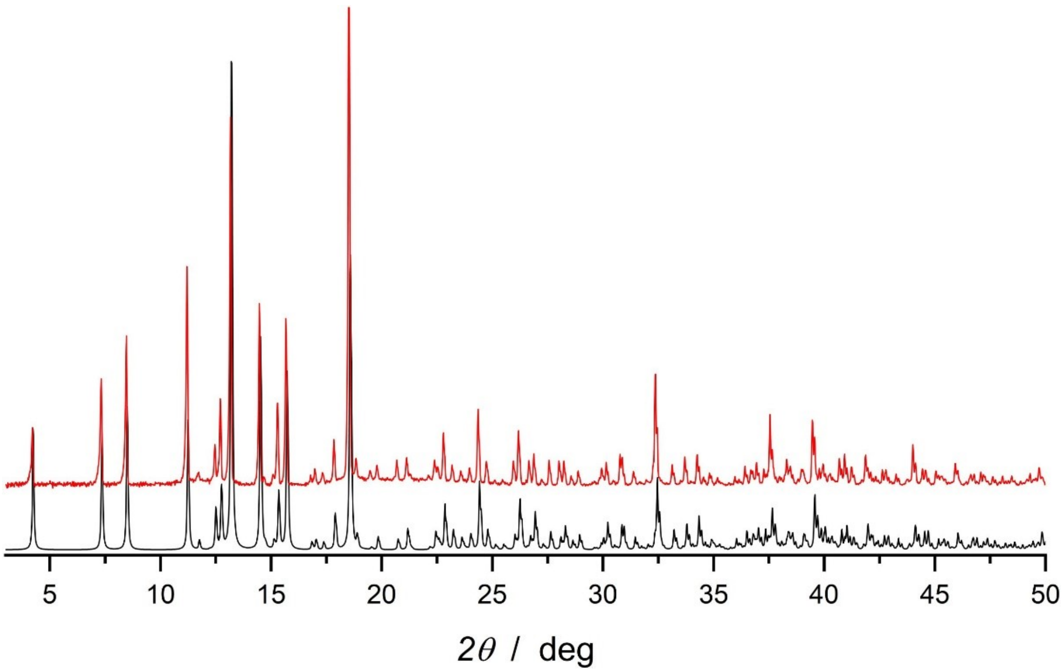

Supplementary Fig. 4. Experimental (red line) and simulated (black line) powder X-ray diffraction experiments for **ErRe₃**. The simulated pattern is calculated based on the 270 K single crystal X-ray diffraction structural model and includes the contribution of the Cu Kβ which is observed in the experimental pattern.

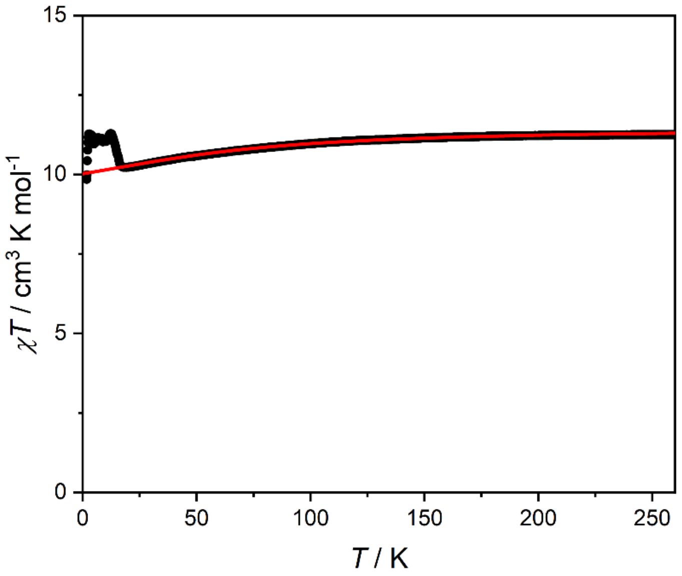

Supplementary Fig. 5. Temperature dependence of the molar magnetic susceptibility and temperature product $\chi T(T)$ for **$ErRe_3$** recorded at $\mu_0 H$ = 0.1 T (black points). The solid red line presents the $\chi T(T)$ obtained from the *ab initio* calculations (see text for details). The observed anomaly below 20 K is caused by the magnetization blocking and the ferromagnetic interactions between the neighbouring $ErRe_3$ molecules.

Supplementary Table 2. Results of the generalized Debye model fitting of the frequency dependence of the AC magnetic susceptibility χ' and χ'' for **$ErRe_3$** recorded at zero magnetic field in the 2.4 – 27 K range presented in Figure 2C (main text).

***T* / K**	**χ_S / cm³ mol⁻¹**	**χ_S error**	**χ_T / cm³ mol⁻¹**	**χ_T error**	***τ* / s**	***τ* error**	***α***	***α* error**	***R*²**
**2.4**	1.277	0.050	4.601	0.019	0.00584	1.6E-4	0.206	0.012	0.99963
**2.7**	1.195	0.049	4.186	0.018	0.00576	1.7E-4	0.202	0.013	0.99958
**3.0**	1.023	0.044	3.803	0.018	0.00533	1.6E-4	0.218	0.013	0.99946
**3.5**	0.987	0.036	3.669	0.015	0.00493	1.2E-4	0.208	0.011	0.99961
**4.0**	0.845	0.046	3.346	0.023	0.00496	1.8E-4	0.240	0.016	0.99904
**4.5**	0.764	0.039	2.937	0.018	0.00469	1.6E-4	0.234	0.015	0.99917
**5.0**	0.703	0.032	2.612	0.014	0.00442	1.4E-4	0.225	0.014	0.99932
**5.5**	0.653	0.029	2.361	0.012	0.00419	1.3E-4	0.217	0.014	0.99937
**6.0**	0.616	0.026	2.161	0.011	0.00398	1.2E-4	0.206	0.014	0.99941
**6.5**	0.513	0.019	1.816	0.008	0.00323	8.5E-5	0.191	0.012	0.99947
**7.0**	0.489	0.017	1.680	0.007	0.00296	7.3E-5	0.172	0.012	0.99954
**7.5**	0.464	0.015	1.556	0.005	0.00267	6.2E-5	0.153	0.011	0.99962
**8.0**	0.420	0.013	1.454	0.005	0.00231	5.0E-5	0.146	0.010	0.99958
**8.5**	0.397	0.011	1.364	0.003	0.00206	4.0E-5	0.130	0.009	0.99970
**9.0**	0.363	0.010	1.287	0.003	0.00178	3.1E-5	0.124	0.008	0.99970
**9.5**	0.343	0.009	1.220	0.003	0.00158	2.7E-5	0.114	0.008	0.99975
**10.0**	0.317	0.007	1.160	0.002	0.00137	2.0E-5	0.108	0.007	0.99977
**10.5**	0.289	0.009	1.051	0.005	0.0012	2.1E-5	0.083	0.011	0.99936
**11.0**	0.278	0.010	0.985	0.005	0.00103	2.1E-5	0.059	0.013	0.99900
**11.5**	0.265	0.009	0.946	0.005	9.13E-4	1.7E-5	0.052	0.012	0.99914
**12.0**	0.237	0.006	0.913	0.003	7.89E-4	1.0E-5	0.072	0.008	0.99949
**12.5**	0.218	0.008	0.881	0.004	7.12E-4	1.4E-5	0.081	0.012	0.99879
**13.0**	0.216	0.007	0.835	0.004	6.14E-4	1.0E-5	0.055	0.011	0.99904
**13.5**	0.203	0.008	0.815	0.004	5.54E-4	1.1E-5	0.060	0.012	0.99892
**14.0**	0.205	0.007	0.778	0.003	4.97E-4	8.3E-6	0.031	0.011	0.99919
**14.5**	0.194	0.007	0.751	0.003	4.43E-4	8.0E-6	0.033	0.011	0.99923
**15.0**	0.183	0.007	0.729	0.003	3.98E-4	7.4E-6	0.036	0.011	0.99910
**15.5**	0.170	0.006	0.703	0.003	3.49E-4	6.0E-6	0.038	0.011	0.99894
**16.0**	0.160	0.004	0.691	0.002	3.16E-4	4.3E-6	0.052	0.008	0.99931
**16.5**	0.156	0.004	0.666	0.002	2.83E-4	3.2E-6	0.040	0.007	0.99950
**17.0**	0.151	0.003	0.647	0.002	2.55E-4	2.6E-6	0.038	0.006	0.99962
**17.5**	0.149	0.003	0.627	0.002	2.31E-4	2.5E-6	0.025	0.007	0.99960
**18.0**	0.140	0.004	0.608	0.002	2.05E-4	2.8E-6	0.031	0.008	0.99935
**18.5**	0.138	0.003	0.593	0.001	1.86E-4	1.9E-6	0.023	0.006	0.99966
**19.0**	0.131	0.002	0.577	0.001	1.64E-4	1.3E-6	0.025	0.005	0.99966
**19.5**	0.126	0.002	0.565	0.001	1.47E-4	1.2E-6	0.027	0.005	0.99971
**20.0**	0.121	0.002	0.551	0.001	1.30E-4	8.8E-7	0.028	0.004	0.99974
**20.5**	0.120	0.002	0.534	0.001	1.16E-4	1.2E-6	0.014	0.007	0.99944
**21.0**	0.115	0.002	0.525	0.001	1.03E-4	7.4E-7	0.022	0.005	0.99974
**21.5**	0.114	0.002	0.511	0.001	9.04E-5	8.4E-7	0.013	0.006	0.99961
**22.0**	0.112	0.004	0.498	0.001	8.0E-5	1.2E-6	0.007	0.009	0.99907
**22.5**	0.110	0.004	0.488	0.001	7.04E-5	1.0E-6	0.005	0.009	0.99914
**23.0**	0.106	0.002	0.480	0.001	6.17E-5	4.8E-7	0.011	0.005	0.99978
**23.5**	0.102	0.005	0.471	0.001	5.41E-5	9.6E-7	0.013	0.011	0.99906
**24.0**	0.102	0.003	0.460	0.001	4.71E-5	5.6E-7	0.004	0.007	0.99961
**24.5**	0.100	0.006	0.449	0.001	4.07E-5	8.7E-7	0.000	0.012	0.99898
**25.0**	0.099	0.006	0.441	0.001	3.54E-5	7.9E-7	0.000	0.012	0.99910
**25.5**	0.097	0.006	0.435	0.001	3.08E-5	7.2E-7	0.004	0.012	0.99926
**26.0**	0.096	0.007	0.425	0.001	2.64E-5	7.5E-7	0.000	0.014	0.99917
**26.5**	0.094	0.008	0.418	0.001	2.27E-5	6.7E-7	0.000	0.013	0.99938
**27.0**	0.093	0.008	0.411	0.001	1.96E-5	6.0E-7	0.000	0.012	0.99955

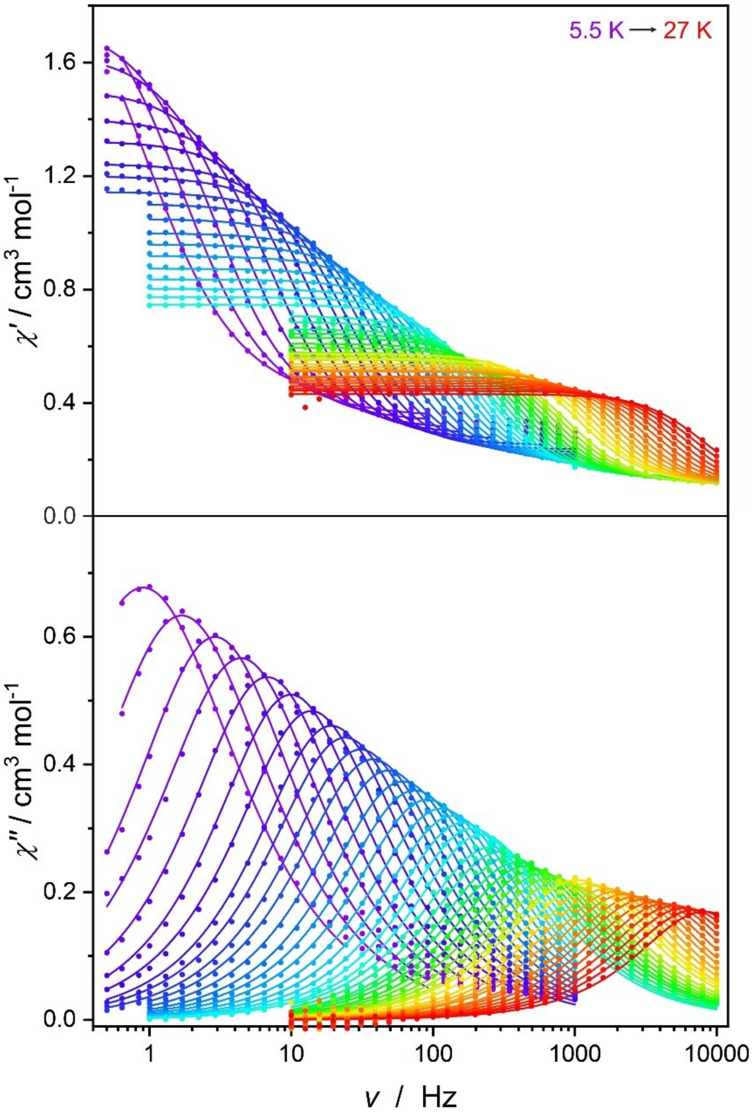

Supplementary Fig. 6. Frequency dependence of the AC magnetic susceptibility χ' and χ'' for **$ErRe_3$** recorded at H_{DC} = 0.15 T in the 5.5 – 27 K range using Quantum Design MPMS3 and PPMS instruments. Solid lines are the best fits to the generalized Debye model. The relevant fitting parameters can be found in Supplementary Table 3 below.

Supplementary Table 3. Results of the generalized Debye model fitting of the frequency dependence of the AC magnetic susceptibility χ' and χ'' for **$ErRe_3$** recorded at H_{DC} = 0.15 T in the 5.5 – 27 K range presented in Supplementary Fig. 6.

T / K	χ_S / cm³ mol⁻¹	χ_S error	χ_T / cm³ mol⁻¹	χ_T error	τ / s	τ error	α	α error	R^2
**5.5**	0.382	0.004	2.201	0.019	0.1769	0.0033	0.185	0.006	0.99954
**6.0**	0.348	0.004	1.949	0.013	0.0943	0.0013	0.148	0.006	0.99935
**6.5**	0.330	0.004	1.749	0.007	0.0549	5.1E-4	0.106	0.005	0.99950
**7.0**	0.302	0.004	1.644	0.005	0.0362	3.1E-4	0.107	0.005	0.99959
**7.5**	0.287	0.003	1.507	0.003	0.0235	1.5E-4	0.081	0.004	0.99976
**8.0**	0.269	0.003	1.403	0.003	0.0160	1.1E-4	0.068	0.004	0.99973
**8.5**	0.250	0.003	1.325	0.003	0.01151	7.9E-5	0.067	0.004	0.99971
**9.0**	0.235	0.003	1.244	0.003	0.00831	6.5E-5	0.058	0.005	0.99961
**9.5**	0.224	0.002	1.200	0.002	0.00663	4.7E-5	0.063	0.004	0.99970
**10.0**	0.214	0.002	1.145	0.002	0.00517	3.5E-5	0.060	0.004	0.99974
**10.5**	0.206	0.002	1.103	0.002	0.00423	2.5E-5	0.060	0.004	0.99979
**11.0**	0.198	0.002	1.050	0.002	0.00331	1.9E-5	0.055	0.004	0.99981
**11.5**	0.190	0.002	0.999	0.001	0.00261	1.3E-5	0.051	0.003	0.99987
**12.0**	0.184	0.002	0.959	0.001	0.00214	1.0E-5	0.048	0.003	0.99989
**12.5**	0.177	0.002	0.918	0.001	0.00174	8.0E-6	0.046	0.003	0.99990
**13.0**	0.171	0.002	0.874	0.001	0.00140	6.4E-6	0.038	0.003	0.99991
**13.5**	0.167	0.002	0.834	0.001	0.00116	5.3E-6	0.030	0.003	0.99991
**14.0**	0.164	0.002	0.802	0.001	9.81E-4	4.4E-6	0.021	0.003	0.99992
**14.5**	0.161	0.002	0.773	0.001	8.42E-4	3.7E-6	0.014	0.003	0.99993
**15.0**	0.157	0.002	0.747	0.001	7.25E-4	3.4E-6	0.010	0.003	0.99992
**15.5**	0.139	0.002	0.708	0.002	6.19E-4	5.5E-6	0.032	0.006	0.99935
**16.0**	0.136	0.002	0.686	0.002	5.35E-4	5.0E-6	0.027	0.006	0.99929
**16.5**	0.134	0.002	0.660	0.002	4.59E-4	4.5E-6	0.019	0.006	0.99923
**17.0**	0.129	0.002	0.646	0.003	4.05E-4	3.6E-6	0.029	0.006	0.99942
**17.5**	0.125	0.002	0.631	0.003	3.53E-4	3.0E-6	0.031	0.005	0.99949
**18.0**	0.123	0.002	0.610	0.003	3.06E-4	3.2E-6	0.023	0.007	0.99920
**18.5**	0.120	0.002	0.595	0.002	2.68E-4	2.7E-6	0.024	0.006	0.99929
**19.0**	0.117	0.003	0.578	0.002	2.32E-4	3.2E-6	0.020	0.009	0.99877
**19.5**	0.114	0.002	0.567	0.002	2.03E-4	2.1E-6	0.024	0.007	0.99930
**20.0**	0.110	0.002	0.553	0.001	1.77E-4	1.7E-6	0.027	0.006	0.99947
**20.5**	0.108	0.003	0.543	0.002	1.54E-4	1.9E-6	0.031	0.008	0.99914
**21.0**	0.106	0.003	0.526	0.001	1.31E-4	1.5E-6	0.025	0.007	0.99926
**21.5**	0.106	0.005	0.508	0.002	1.11E-4	2.2E-6	0.008	0.013	0.99790
**22.0**	0.103	0.004	0.498	0.002	9.53E-5	1.7E-6	0.012	0.011	0.99845
**22.5**	0.100	0.004	0.488	0.001	8.21E-5	1.2E-6	0.013	0.009	0.99908
**23.0**	0.096	0.004	0.483	0.001	7.11E-5	1.0E-6	0.025	0.009	0.99923
**23.5**	0.097	0.007	0.466	0.002	5.99E-5	1.7E-6	0.008	0.017	0.99729
**24.0**	0.093	0.003	0.462	0.001	5.17E-5	7.0E-7	0.022	0.008	0.99949
**24.5**	0.089	0.005	0.453	0.001	4.35E-5	8.1E-7	0.027	0.010	0.99923
**25.0**	0.089	0.007	0.444	0.002	3.70E-5	9.9E-7	0.022	0.014	0.99875
**25.5**	0.088	0.007	0.435	0.001	3.16E-5	8.9E-7	0.016	0.014	0.99892
**26.0**	0.087	0.008	0.427	0.001	2.67E-5	8.0E-7	0.016	0.014	0.99912
**26.5**	0.084	0.010	0.419	0.001	2.26E-5	8.8E-7	0.017	0.016	0.99899
**27.0**	0.087	0.016	0.407	0.002	1.95E-5	1.2E-6	0.000	0.025	0.99802

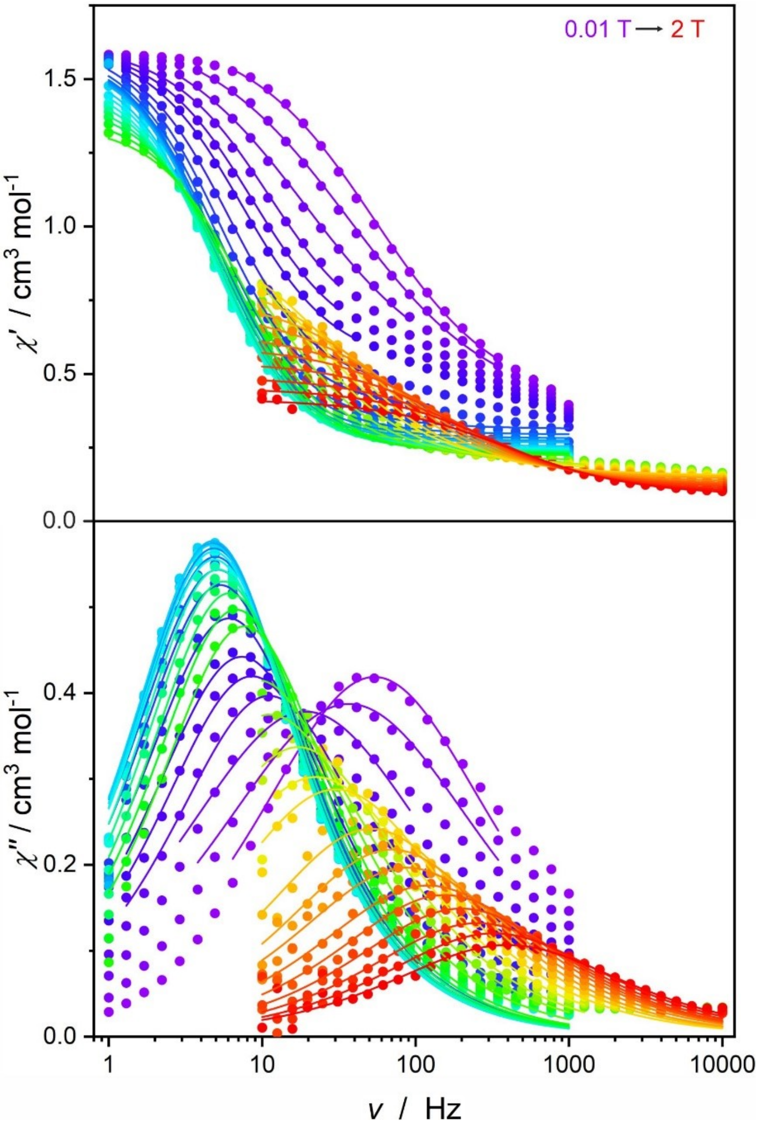

Supplementary Fig. 7. Frequency dependence of the AC magnetic susceptibility χ' and χ'' for **$ErRe_3$** recorded at T = 7 K in the 0.01 – 2 T range using Quantum Design MPMS3 and PPMS instruments. Solid lines are the best fits to the generalized Debye model. The relevant fitting parameters can be found in Supplementary Table 4 below.

Supplementary Table 4. Results of the generalized Debye model fitting of the frequency dependence of the AC magnetic susceptibility *χ*' and *χ*'' for **ErRe₃** recorded at *T* = 7 K in the 0.01 – 2 T range presented in Supplementary Fig. 6.

***H* / T**	**χ_S / cm³ mol⁻¹**	**χ_S error**	**χ_T / cm³ mol⁻¹**	**χ_T error**	***τ* / s**	***τ* error**	***α***	***α* error**	***R*²**
**0.01**	0.371	0.014	1.677	0.013	0.00295	7.1E-5	0.274	0.012	0.99924
**0.02**	0.352	0.013	1.700	0.013	0.00433	1.1E-4	0.336	0.012	0.99931
**0.03**	0.442	0.019	1.700	0.017	0.00828	2.6E-4	0.311	0.016	0.99933
**0.04**	0.583	0.018	1.628	0.010	0.01433	4.1E-4	0.173	0.016	0.99942
**0.05**	0.559	0.014	1.630	0.010	0.01855	4.1E-4	0.154	0.014	0.99939
**0.06**	0.517	0.012	1.630	0.012	0.02158	4.3E-4	0.145	0.013	0.99916
**0.08**	0.442	0.008	1.624	0.011	0.02617	4.2E-4	0.123	0.010	0.99914
**0.10**	0.374	0.008	1.636	0.013	0.02972	5.6E-4	0.115	0.011	0.99835
**0.12**	0.313	0.006	1.625	0.017	0.03095	7.5E-4	0.119	0.012	0.99673
**0.14**	0.294	0.006	1.606	0.016	0.03208	7.1E-4	0.102	0.012	0.99696
**0.16**	0.281	0.006	1.598	0.016	0.03283	7.0E-4	0.093	0.011	0.99700
**0.18**	0.273	0.006	1.588	0.017	0.03329	7.3E-4	0.086	0.012	0.99670
**0.20**	0.266	0.006	1.579	0.017	0.03332	7.4E-4	0.082	0.012	0.99647
**0.25**	0.254	0.004	1.560	0.011	0.03379	4.9E-4	0.083	0.008	0.99849
**0.30**	0.247	0.004	1.538	0.010	0.03303	4.4E-4	0.085	0.007	0.99875
**0.35**	0.243	0.004	1.515	0.010	0.03268	4.3E-4	0.086	0.007	0.99875
**0.40**	0.239	0.004	1.486	0.010	0.03076	4.6E-4	0.087	0.008	0.99841
**0.45**	0.235	0.004	1.454	0.011	0.02849	4.5E-4	0.089	0.009	0.99818
**0.50**	0.231	0.005	1.425	0.011	0.02604	4.4E-4	0.092	0.009	0.99802
**0.55**	0.227	0.005	1.391	0.011	0.02350	4.2E-4	0.099	0.010	0.99782
**0.60**	0.222	0.005	1.355	0.011	0.02073	3.9E-4	0.108	0.010	0.99765
**0.70**	0.205	0.003	1.275	0.032	0.01686	8.6E-4	0.160	0.013	0.99699
**0.80**	0.188	0.004	1.242	0.029	0.01419	7.2E-4	0.213	0.013	0.99725
**0.90**	0.183	0.003	1.119	0.016	0.00927	3.0E-4	0.205	0.010	0.99817
**1.00**	0.170	0.002	1.057	0.010	0.00710	1.5E-4	0.239	0.006	0.99935
**1.10**	0.154	0.004	1.004	0.018	0.00527	2.4E-4	0.242	0.015	0.99480
**1.20**	0.149	0.003	0.847	0.008	0.00310	7.8E-5	0.229	0.010	0.99785
**1.30**	0.142	0.003	0.769	0.007	0.00227	5.3E-5	0.227	0.010	0.99800
**1.40**	0.132	0.003	0.717	0.005	0.00182	3.9E-5	0.246	0.009	0.99842
**1.50**	0.124	0.002	0.644	0.004	0.00132	2.6E-5	0.243	0.009	0.99863
**1.60**	0.119	0.003	0.597	0.005	0.00103	2.6E-5	0.230	0.012	0.99763
**1.70**	0.113	0.004	0.543	0.005	8.03E-4	2.5E-5	0.228	0.014	0.99670
**1.80**	0.104	0.004	0.493	0.004	6.29E-4	2.1E-5	0.239	0.015	0.99656
**1.90**	0.095	0.004	0.454	0.004	4.90E-4	1.7E-5	0.248	0.015	0.99668
**2.00**	0.085	0.004	0.417	0.003	4.01E-4	1.4E-5	0.272	0.015	0.99704

Supplementary Table 5. The employed basis sets for *ab initio* calculations.

Basis set 1	Basis set 2
Er.ANO-RCC-VDZP<br>Re.ANO-RCC-VDZ<br>C.ANO-RCC-VDZ<br>H.ANO-RCC-VDZ	Er.ANO-RCC-VTZP<br>Re.ANO-RCC-VDZP<br>C.ANO-RCC-VDZP<br>H.ANO-RCC-VDZ

Supplementary Table 6. Spin-orbit energies (cm^{-1}) and the *g*-tensors of the lowest in energy Kramers doublets.

Multiplet		Basis set 1	Basis set 2
1		0<br>0	0<br>0
2		155.3678<br>155.3678	154.0022<br>154.0022
3		252.3421<br>252.3421	251.7256<br>251.7256
4		330.1533<br>330.1533	330.4627<br>330.4627
5		400.1957<br>400.1957	400.5357<br>400.5357
6		482.2936<br>482.2936	484.0622<br>484.0622
7		536.0058<br>536.0058	536.8215<br>536.8215
8		567.7605<br>567.7605	568.1037<br>568.1037
**Multiplet**			
1	g_x=<br>g_y=<br>g_z=	0.000174<br>0.000394<br>17.905279	0.000254<br>0.000486<br>17.905174
2	g_x=<br>g_y=<br>g_z=	0.044452<br>0.045268<br>15.501484	0.050255<br>0.051285<br>15.500554
3	g_x=<br>g_y=<br>g_z=	0.027204<br>0.095619<br>13.092686	0.020446<br>0.097873<br>13.089441
4	g_x=<br>g_y=<br>g_z=	0.165059<br>0.286776<br>10.589177	0.176105<br>0.296341<br>10.573533
5	g_x=<br>g_y=<br>g_z=	4.367102<br>4.803154<br>7.248873	4.578907<br>5.038692<br>7.139900

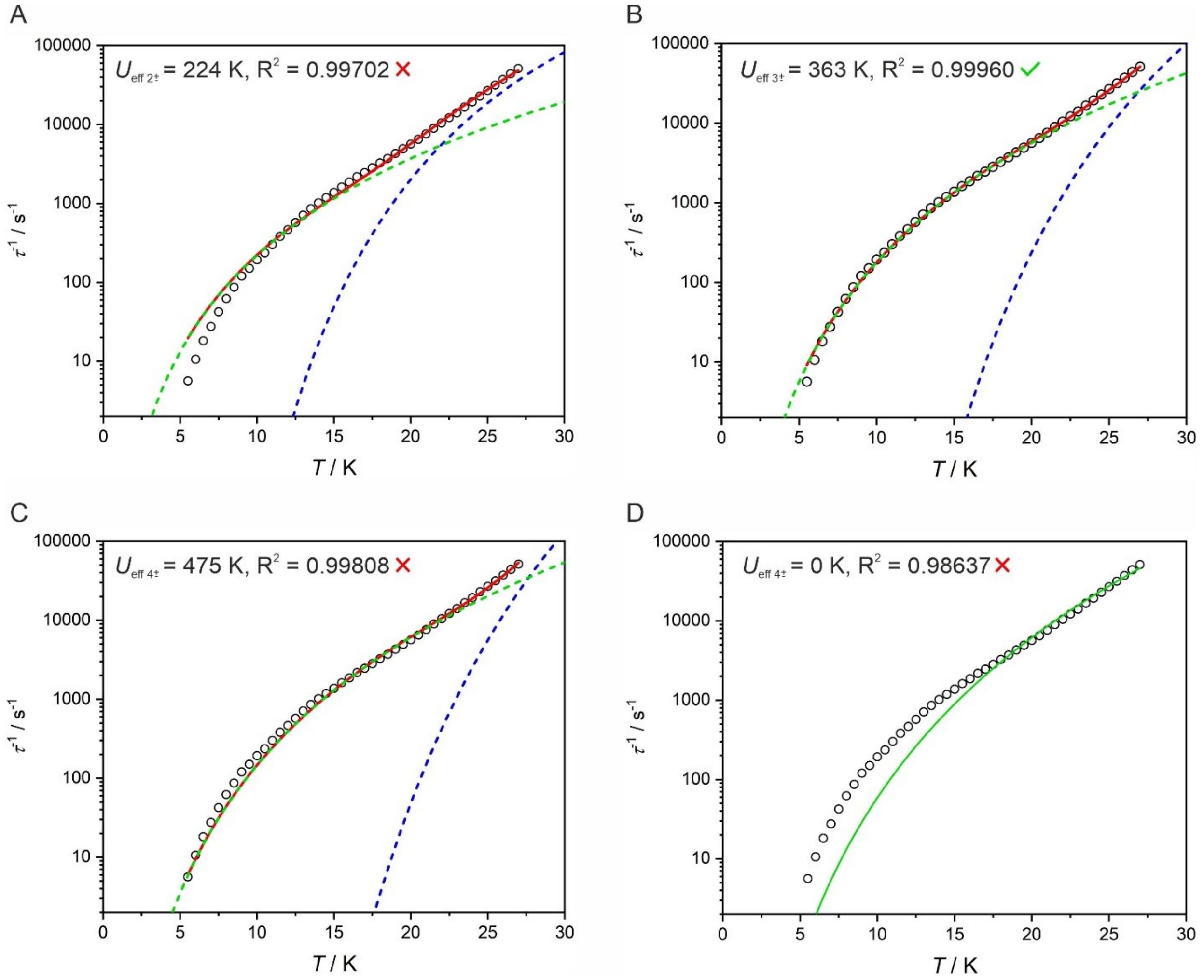

Supplementary Fig. 8. Temperature dependence of the magnetic relaxation rate $\tau^{-1}(T)$ for **$ErRe_3$** under applied 0.15 T DC magnetic field (circles). Solid lines represent the best fits assuming Raman-like and Orbach relaxation processes with *C*, n, τ_0 as free parameters and U_{eff} fixed at 224 K (A), 363 K (B), 475 K (C) or Raman-like relaxation process only (D). Fitting presented in B shows highest R^2 and is assumed to provide the most accurate value of the energy barrier for magnetization reversal. Fitting assuming Raman-like relaxation only (D) shows the lowest R^2 which sanctions the need for including the Orbach mechanism.

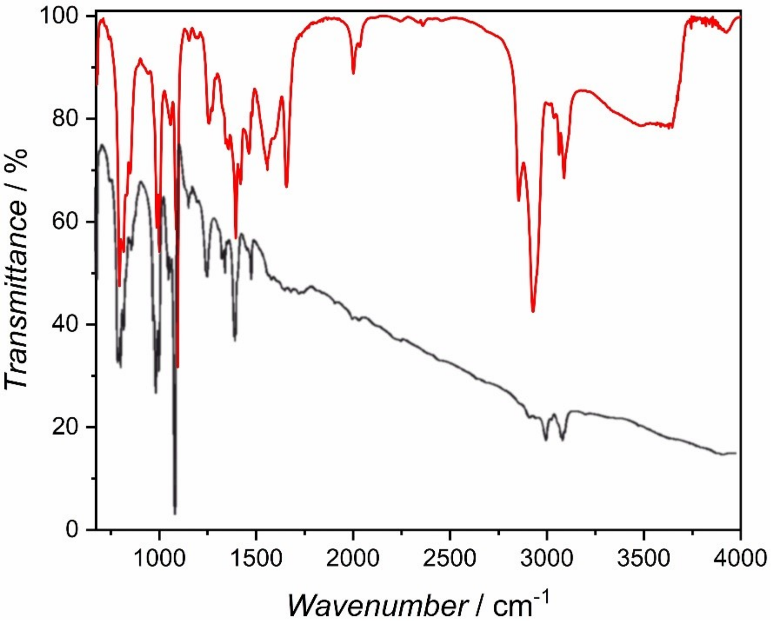

Supplementary Fig. 9. IR spectra of crushed crystals of **$ErRe_3$** (red solid line) with the overlaid IR spectrum of **$SmRe_3$** recorded as KBr pellet (black solid line) reproduced from Ref. 10 in the 675-4000 cm^{-1} range (the IR spectrum from Fig. S6 from the supplementary information of Ref. 10 was traced using CorelDRAW 2019 software). Both spectra are very similar. Weak Re-H stretching modes at 2001 and 2034 cm^{-1} observed for **$ErRe_3$** are due to the slight decomposition of this extremely sensitive compound. Note, that these stretches are also present in the IR spectrum of **$SmRe_3$,** but are largely obstructed by a significant background reaching 40% transmittance at 2000 cm^{-1} and nearly 15% transmittance at 4000 cm^{-1}.

Supplementary references

1. Sheldrick GM. Crystal structure refinement with SHELXL. *Acta Crystallogr. C*, *71*, 3–8 (2015)

2. Dolomanov OV, Bourhis LJ, Gildea RJ, Howard JAK, Puschmann H. OLEX2: a complete structure solution, refinement and analysis program. *J. Appl. Crystallogr.* **42**, 339–341 (2009)

3. Ren M, Pinkowicz D, Yoon Y, Kim K, Zheng L-M, Breedlove BK, Yamashita M. Dy(III) Single-Ion Magnet Showing Extreme Sensitivity to (De)hydration. *Inorg. Chem.* **52**, 8342-8348 (2013)

4. Aquilante F, Autschbach J, Baiardi A, Battaglia S, Borin VA, Chibotaru LF, Conti I, Vico LD, Delcey M, Galván IF, Ferré N, Freitag L, Garavelli M, Gong X, Knecht S, Larsson ED, Lindh R, Lundberg M, Malmqvist PÅ, Nenov A, Norell J, Odelius M, Olivucci M, Pedersen TB, Pedraza-González L, Phung QM, Pierloot K, Reiher M, Schapiro I, Segarra-Martí J, Segatta F, Seijo L, Sen S, Sergentu D-C, Stein CJ, Ungur L, Vacher M, Valentini A, Veryazov V. Modern quantum chemistry with [Open]Molcas. *J. Chem. Phys.* **152**, 214117 (2020)

5. Roos BO, Taylor PR, Sigbahn PEM. A complete active space SCF method (CASSCF) using a density matrix formulated super-CI approach. *Chem. Phys.* **48**, 157–173 (1980)

6. Malmqvist PÅ, Roos BO, Schimmelpfennig B. The restricted active space (RAS) state interaction approach with spin-orbit coupling. *Chem. Phys. Lett.* **357**, 230–240 (2002)

7. Chibotaru LF, Ungur L. Ab initio calculation of anisotropic magnetic properties of complexes. I. Unique definition of pseudospin Hamiltonians and their derivation. *J. Chem. Phys.* 137, 064112 (2012)

8. Ungur L, Chibotaru LF. in *Lanthanides and actinides in molecular magnetism* (eds. R. A. Layfield, M. Murugesu) 153–184 (Wiley-VCH Verlag GmbH & Co. KGaA) (2015)

9. Ungur L, Thewissen M, Costes J-P, Wernsdorfer W, Chibotaru LF. Interplay of strongly anisotropic metal ions in magnetic blocking of complexes. *Inorg. Chem.* **52**, 6328–6337 (2013)

10. Butovskii MV, Döring C, Bezugly V, Wagner FR, Grin Y, Kempe R. Molecules containing rare-earth atoms solely bonded by transition metals. *Nature Chemistry* 2, 741-744 (2010)
